# Genetically Encoded Calcium Indicators: A New Tool in Renal Hypertension Research

**DOI:** 10.3389/fmed.2019.00128

**Published:** 2019-06-13

**Authors:** Cheng Zhong, Johanna Schleifenbaum

**Affiliations:** Institute of Vegetative Physiology, Charité–Universitätsmedizin Berlin, Corporate Member of Freie Universität Berlin, Humboldt-Universität zu Berlin, Berlin, Germany

**Keywords:** calcium, GCaMP, hypertension, kidney, imaging

## Abstract

Hypertension is ranked as the third cause of disability-adjusted life-years. The percentage of the population suffering from hypertension will continue to increase over the next years. Renovascular disease is one of the most common causes of secondary hypertension. Vascular changes seen in hypertension are partially based on dysfunctional calcium signaling. This signaling can be studied using calcium indicators (loading dyes and genetically encoded calcium indicators; *GECIs*). Most progress in development has been seen in GECIs, which are used in an increasing number of publications concerning calcium signaling in vasculature and the kidney. The use of transgenic mouse models expressing GECIs will facilitate new possibilities to study dysfunctional calcium signaling in a cell type-specific manner, thus helping to identify more specific targets for treatment of (renal) hypertension.

## Introduction

Hypertension, a disease with high incidence and a leading risk factor of mortality, is ranked as the third cause of disability-adjusted life-years (1). In particular, raised blood pressure is an important risk factor for cardiovascular diseases and chronic kidney disease. Worldwide, the estimated total number of adults with hypertension in 2000 was 972 million (26.4% of the adult population) (2). The number of adults with raised blood pressure increased to 1.13 billion in 2015, with the increase occurring largely in low-income and middle-income countries (3). The number of adults with hypertension in 2,025 is predicted to increase to a total of 1.56 billion (1.54–1.58 billion) (2). The most common causes of secondary, non-essential hypertension are renovascular disease, intrinsic renal disease, and primary hyperaldosteronism (4). These data show that hypertension is a global burden.

Calcium is one of the most important and multi-functional second messengers in cell biology, not only controlling contraction of the striated (5) and vascular smooth muscle, but also regulating cellular processes such as growth, proliferation, transcription, exocytosis, and apoptosis (6). Additionally, regulation of thick myofilament Ca^2+^ sensitivity, cytosolic Ca^2+^ induces conformational changes of thin filaments, which together determine actin-myosin and myocyte relaxation or constriction (7). Vascular smooth muscle cell (VSMC) contraction is initiated by an increase in the global intracellular calcium ([Ca^2+^]i) concentration, which is caused by an opening of voltage-gated calcium channels, particularly L-type Ca_V_1.2 channels (8). Importantly, the VSMCs' phenotype can switch in cardiovascular disease. For example, de-differentiation of the VSMCs is associated with a shift of the expression of Ca^2+^ channels from voltage-gated to voltage-insensitive Ca^2+^ channels (9). In addition, Ca^2+^ entry in non-contractile (dedifferentiated) VSMCs occurs predominantly *via* store-operated Ca^2+^ entry (SOCE) and receptor-operated Ca^2+^ entry (ROCE) pathways. These changes may have physiological importance for normal smooth muscle function and may influence VSMC behavior under pathophysiological conditions (9). With age, reactivity of small arteries is lowered. This is followed by alterations of arterial stiffness, arterial wall thickening, and a reduced myogenic responsiveness, thus increasing total peripheral resistance (10, 11). Calcium antagonists (e.g., L-type channel inhibitors) are often used as anti-hypertensive drugs. Since the reasons for dedifferentiation of VSMCs are poorly understood (12), it is important to identify calcium signaling pathways in VSMCs in hypertension and cardiovascular disease and determine their functions, which may ultimately lead to new drug targets.

## Calcium Indicators: Loading Dyes, Fusion Proteins, and Genetically Modified GECIs

Calcium imaging of living tissue has turned out to be a useful tool for the investigation of different intracellular calcium signals. Fluorescent free calcium-binding dyes such as Fura-2 enabled a first visualization of intracellular calcium upon loading of the tissue (13). Fura-2 is still one of the most frequently used ratiometric Ca^2+^ indicators. It is excited at two different wavelengths. The Ca^2+^ unbound form of Fura-2 is excited at 380 nm and the Ca^2+^ bound form at 340 nm. The emitted light is measured at around 510 nm (14). Thus, the intensity of the emitted fluorescence light changes depending on the calcium ion concentration, while a high spatial resolution can be reached. Although low temporal resolution can be a problem for recording of fast Ca^2+^ transients, such as Ca^2+^ sparks in VSMCs (14, 15), a major disadvantage of loading dyes such as Fura-2 is a possible uneven tissue distribution of the dye as well as loading of cell structures not intended for investigation (16).

Genetically encoded Ca^2+^ indicators (GECIs) show a huge progress for imaging to solve poor selectivity and disadvantages of loading dyes into the cytosol before measurement (**Table 2**). The first protein-based Ca^2+^ indicator was photoprotein aequorin, purified from jellyfish Aequorea Victoria, injected into cells in the early 1970s (17). After cloning of its cDNA, recombinant aequorin became the most frequently used probe to measure intracellular Ca^2+^. Another important step was the advancement of a green fluorescent protein (GFP), which enabled the investigation of the spatio-temporal distribution of proteins in living cells, through the formation of fused protein-GFP structures. This coupling meant a co-expression of GFP upon target protein expression, thus marking the protein via fluorescence (18–21) (Table 1). GECIs are subject to photobleaching after extended excitation, although usually less than loading dyes (29). Thus, complex photobleaching curves have to be considered when analyzing obtained data. The probe insensitivity to Mg^2+^ is an important issue, because changes of Mg^2+^ concentration can trigger cell activation. Inaccurate intracellular location is also another issue to be solved using GECIs. A kind of larger dynamic range, robust photonic and thermal stability Ca^2+^ probe is needed. (30) Furthermore, oligomerization is another adverse property of GFP. Therefore, mutagenesis approaches are needed to recover the functional expression of monomeric forms of GFP (31) (Tables 2, 3).

**Table 1:** Classes of genetically encoded calcium indicators (GECIs).

**Class**	**Composition**	**References**
1	- Bioluminescent reporters based on aequorin photoprotein (e.g., *GFP*) - Light generated by a chemical reaction requiring reconstitution of the indicator with a co-factor	(22, 23)
2	- Based on single fluorescent proteins (e.g., *GCaMP*) - Calcium-responsive elements as calmodulin (CaM) (or parts of it) inserted into fluorescent protein; calcium binding alters protonation state, conformation, and spectral chromophore properties	(24, 25)
3	- “Cameleon”-type - Calcium-responsive elements between two fluorescent proteins; calcium binding alters efficiency of fluorescence resonance energy transfer (FRET)	(26–28)

**Table 2:** Advantages of genetically encoded indicators (GECIs).

**Major advantages**	**Explanation**	**Reference**
Accurate location	Monitoring activity among genetically defined subsets of cells, i.e., targeted to specific cell types	(32)
Dynamics	Measuring calcium dynamics in specific subcellular compartments	
Long-term	Long-term calcium imaging *in vivo* and imaging in a relatively non-invasive manner	

**Table 3:** Limitations of genetically encoded indicators (GECIs).

**Major limitations**	**Explanation**	**References**
Sensor signal strength	1. Chameleons' molecules exhibit limited *in vivo* signal strength 2. Endogenous calmodulin would decrease probe signal strength 3. Low expression: often resulting in sensors' inactivation or reduced dynamic range	(30, 33, 34)
Stability	Thermal instability: temperature-dependent structure shifts may markedly alter fluorescent properties resulting in poor visualization or signally properties in *in vivo* experiments	(35)
Transition kinetics	Change of Ca^2+^ concentration affects fluorescence on and off rates; limited sensitivity and slow response kinetics	(34, 36, 37)
Interaction between the sensor and cellular molecules	Covalent modulation of calmodulin or binding to endogenous targets will be eliminated; can be susceptible to effects of Ca^2+^ buffering	(34, 38)

**Figure 1:**
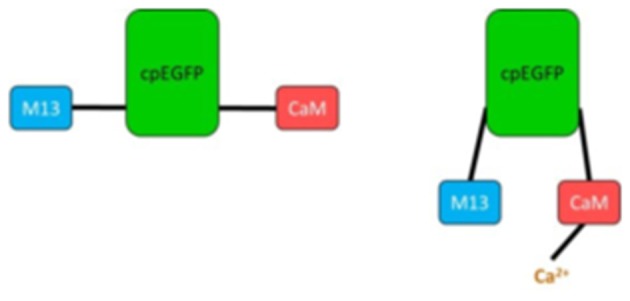
Simplified scheme of GCaMP unbound **(Left)** and bound to Ca^2+^ **(Right)**. M13, fragment of myosin light chain kinase; cpEGFP, circularly permuted enhanced GFP; CaM, calmodulin.

GFP is used as scaffolding for the most recent fluorescent calcium indicators, namely genetically encoded calcium indicators (GECIs) like GCaMP (26). In those proteins, circularly permuted forms of GFP are fused to calmodulin (CaM) and the M13 domain of the myosin light chain kinase (MLCK). The latter is able to bind CaM. If calcium ions are present, GCaMP changes its conformation due to calcium binding to CaM (Figure 1). This leads to a bright fluorescence through a rapid deprotonation of the chromophore (26).

GECIs can be used not only for Ca^2+^ imaging in the cytosol but also in subcellular compartments, i.e., in a wide spectrum of organelles. Among them, the GCaMP6f probe targeted to mitochondria (4mtGCaMP) has been recently developed to measure mitochondrial calcium levels (39). Other protocols of using GECIs have been developed for Ca^2+^ imaging in the nucleus and endoplasmic reticulum (40, 41). Importantly, the fluorescent indicators' brightness could be problematic for Ca^2+^ imaging in organelles because of the acidic environment which can affect fluorescence; therefore, specific tasks, e.g., “chameleon” type indicators, have been developed for target locations in different organelles to detect Ca^2+^ release (42). The fluorescent indicators mentioned above emit green fluorescence. Notably, there are also modified variants of GFP using different fluorescence spectra. Known fluorescence examples include RFP (red fluorescence) (43), YFP (yellow fluorescence) (44, 45), or CFP (cyan fluorescence) (46, 47). The modifications also provide the basis for GECIs as RCaMP (red fluorescence) (48), YCaMP (yellow fluorescence) (48), or CyCaMP (cyan fluorescence) (48). Different color channels allows for the imaging of tissues already expressing GFP, as well as the reduction of autofluorescence compared to GCaMP or loading dyes.

Genetically encoded calcium indicators (GECIs) can be divided into three different classes (Table 1) (49), two of them have just been described. Class 3 indicators (“chameleon”-type sensors) contain two fluorescent proteins and are applied to measurements involving fluorescent resonance energy transfer (FRET).

## Vascular Expression of GCaMP Indicators

One huge advantage of GCaMP is the possibility of cell type-specific expression. It can be coupled to a promoter only expressed in the cell type of interest, so activation of this promoter leads to co-expression of GCaMP. Many studies involving GCaMP have been done in neuronal tissue (50–52), but vascular applications are possible, for example, using mice expressing GCaMP only in acta 2-positive cells (SMC-specific fluorescence), or in connexin 40 (Cx40)—positive cells (endothelial-specific fluorescence) (53–57). Not only can the expression of the protein be detected (as with GFP), but changes in fluorescence intensity reflecting changes in the calcium concentration can also be detected. If GCaMP is co-expressed with another fluorescent protein using other wavelength spectra, e.g., red fluorescent mCherry, a ratio of those two fluorescent proteins can be used for quantification of the intracellular calcium concentration. But expression levels of transgenes show considerable animal-to-animal variation, complicating the analysis of imaging results, and so linearizing the imaging measurement and background in high pixels is needed (58). As GCaMP sensors are stable at mammalian body temperatures, they can also be used for *in vivo* recordings of calcium signaling. GCaMP molecules have been modified and improved on since their first development, allowing higher spatio-temporal resolution, higher sensitivity, and more rapid off kinetics. GCaMP expression even allows calcium imaging of subcellular structures such as a nucleus, endoplasmic reticulum, and mitochondria (57). There is also a review available specifically on the application of GCaMP in cardiomyocytes (59).

## GCaMP Indicators in Kidney Slices

In the vasculature, the above-mentioned Cx40 serves as an endothelial-specific marker protein, enabling cell type-specific calcium imaging. In the kidney, Cx40 was found to be abundantly expressed in specialized SMCs of the juxtaglomerular apparatus (JGA), namely renin-producing granular cells (60). The cells contribute to the tone of afferent arteriole and control of renin synthesis and release. Both processes rely on calcium signals (61, 62), which are positively correlated with renin secretion (63) without the involvement of voltage-gated Ca^2+^ channels (64). Interestingly, these cells contain, in addition to the secretory granules, contractile proteins, which are arranged in a sublemmal network. A paradoxical (inhibitory) role of intracellular calcium in renin secretion could be explained by an increased tone of this sublemmal network, which might impair the pre-exocytotic access of renin granules to the cell membrane (65).

**Figure 2:**
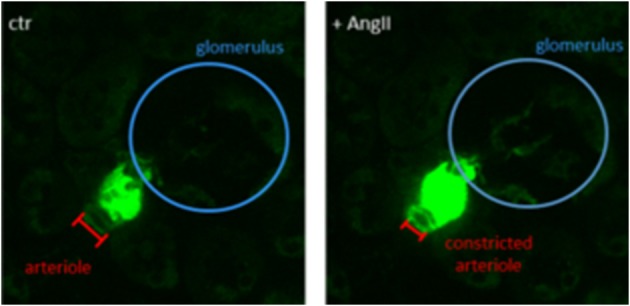
Single frame of a Cx40-GCaMP kidney slice recording. **Left:** Spontaneous calcium transients in Cx40-positive renin-producing granular cells (green). **Right:** Increased calcium signal after treatment with angiotensin II (AngII, 100 nM). Afferent arteriole is constricted upon AngII treatment.

Cx40, a gap junction protein, coordinates propagating signals between individual cells, thus Cx40-positive cells expressing a GCaMP sensor are valuable for studying propagating calcium signals in connected cells and tissues, respectively. Propagating calcium transients can be observed using an *in-situ* kidney slice model of Cx40-GCaMP mice, as shown in Figure 2. The slices already show spontaneous intracellular calcium transients without any treatment. Upon treatment with angiotensin II (AngII), the Ca^2+^ transients are elevated (Figure 2). Characterizing the spontaneous signals and comparing them to AngII-induced transients might be of great interest for (1) studying the control of afferent arteriole tone and renin release and (2) explore potential coupling of the signals regulating both processes.

GCaMP sensors have already been used in the kidney to study calcium signals in podocytes. Pathological changes of the glomerular filtration barrier have been linked to elevated intracellular calcium concentrations in podocytes (66). Signals could be measured *in vivo* using GCaMP sensors in podocin-positive cells (Pod-GCaMP3), providing podocyte-specific fluorescence (67).

## Summary

The ongoing development of genetically modified calcium indicators provides great opportunities for studying calcium signaling in specific cell types and organs linked to the development of hypertension, e.g., in vasculature and kidneys. The improvement of the sensors allows a higher spatio-temporal resolution and a higher sensitivity for qualitative as well as quantitative measurements. Different excitation wavelengths enable simultaneous recordings of calcium signals in different colors, and thus a differentiation of parallel signals in different locations (e.g., SMCs and ECs).

Mice expressing GECIs in a cell type-specific manner can be subject to hypertension-inducing treatments (administration of AngII, or L-NAME), allowing exploration of calcium signaling changes in vasculature and organs such as the kidneys, in comparison to healthy control animals. The effects of different drugs influencing intracellular calcium signaling can be studied *in vivo* as well as *in situ* and can help in finding new therapeutic targets to reverse dysfunctional calcium signaling under hypertensive conditions in vasculature and kidneys. Thus, animal models expressing GCaMP sensors will play an important role in the future of renal hypertension research.

## Author Contributions

All authors listed have made a substantial, direct and intellectual contribution to the work, and approved it for publication.

### Conflict of Interest Statement

The authors declare that the research was conducted in the absence of any commercial or financial relationships that could be construed as a potential conflict of interest. The handling Editor declared shared affiliations, though no other collaboration on this topic or techniques, with both of the authors.
